# Supplementary material for: The smallest known Devonian tetrapod shows unexpectedly derived features
Source: R Soc Open Sci. 2020 Apr 8;7(4):192117. doi: 10.1098/rsos.192117 (PMC7211834; doi:10.1098/rsos.192117)
Supplement: Appendix 1 [file rsos192117supp1.docx]

### APPENDIX 1

### Character list

skull roof, palate

1. Anterior tectal/septomaxilla: anterior tectal (external bone, dorsal to nostril): = 0, septomaxilla (external or internal bone, posterior to nostril) = 1, absent = 2
2. Ectopterygoid /palatine exposure: more or less confined to tooth row = 0, broad mesial exposure additional to tooth row = 1
3. Ectopterygoid reaches subtemporal fossa: no = 0, yes = 1
4. Frontal: absent = 0, present = 1
5. Intertemporal: present = 0, absent = 1
6. Jugal: does not extend anterior to orbit = 0, extends anterior to orbit = 1
7. Lacrimal: contributes to orbital margin = 0, excluded from margin = 1 (Note: this character has been scored as "?" for baphetids because it isn't clear whether the anterior "keyhole" extension of the orbit (see also character 86) should be considered equivalent to the anteroventral margin of the orbit in other taxa.)
8. Lateral rostral present: yes = 0, no = 1
9. Maxilla makes interdigitating suture with vomer: no = 0, yes = 1
10. Maxilla external contact with premaxilla: narrow contact point not interdigitated = 0, interdigitating suture = 1
11. Maxilla extends behind level of posterior margin of orbit: yes = 0, no = 1
12. Median rostral: single = 0, paired = 1, absent = 2
13. Opercular: present = 0, absent = 1
14. Prefrontal: twice as long as broad, or less = 0, three times as long as broad or more = 1
15. Prefrontal: transverse anterior suture with tectal = 0, tapers to point anteriorly = 1
16. Preopercular: present = 0, absent = 1
17. Pterygoids separate in midline = 0, meet in midline anterior to cultriform process = 1
18. Pterygoid quadrate ramus margin in subtemporal fossa: concave = 0, with some convex component = 1
19. Vomers separated by parasphenoid > half length: yes = 0, no = 1
20. Vomers excluded from margin of interpterygoid vacuity: yes = 0, no = 1
21. Vomers nearly as broad as long, or broader = 0, about twice as long as broad, or longer = 1

braincase

1. Basipterygoid process: not strongly projecting with concave anterior face = 0, strongly projecting with flat anterior face = 1
2. Ethmoid: fully ossified = 0, partly or wholly unossified = 1
3. Hypophysial region: solid side wall pierced by small foramina for pituitary vein and other vessels = 0, single large foramen = 1
4. Otic capsule: lateral commissure bearing hyomandibular facets: present = 0, absent = 1
5. Parasphenoid: does not overlap basioccipital = 0, overlaps basioccipital =1
6. Parasphenoid: denticulated field: present = 0, absent = 1
7. Sphenoid: fully ossified, terminating posteriorly in intracranial joint or fused to otoccipital = 0, separated from otoccipital by unossified gap = 1

palatal dentition

1. Ectopterygoid fang pairs: present = 0, absent = 1
2. Ectopterygoid row (3+) of smaller teeth: present = 0, absent = 1
3. Ectopterygoid / palatine shagreen field: absent = 0, present = 1
4. Maxilla tooth number: > 40 = 0, 30-40 = 1, < 30 = 2
5. Palatine row of smaller teeth: present = 0, absent = 1
6. Pterygoid shagreen: dense = 0, a few discontinuous patches or absent = 1
7. Premaxillary tooth proportions: all approximately same size = 0, posteriormost teeth at least twice height of anteriormost teeth = 1
8. Vomerine fang pairs: present = 0, absent = 1
9. Vomerine fang pairs noticeably smaller than other palatal fang pairs: no = 0, yes = 1
10. Vomer anterior wall forming posterior margin of palatal fossa bears tooth row meeting in midline: yes = 0, no = 1
11. Vomerine row of small teeth : present = 0, absent = 1
12. Vomerine shagreen field: absent = 0, present = 1

lower jaw

1. Adductor fossa faces dorsally = 0, mesially = 1
2. Adductor crest: absent = 0, peak anterior to adductor fossa, dorsal margin of fossa concave = 1, peak above anterior part of adductor fossa, dorsal margin of fossa convex = 2
3. Angular – prearticular contact: prearticular contacts angular edge to edge = 0, absent = 1, mesial lamina of angular sutures with prearticular = 2
4. Coronoid (anterior) contacts splenial: no = 0, yes = 1
5. Coronoid (posterior) posterodorsal process: no = 0, yes = 1
6. Coronoid (posterior) posterodorsal process visible in lateral view: no = 0, yes = 1
7. Dentary external to angular + surangular, with chamfered ventral edge and no interdigitations: no = 0, yes = 1
8. Dentary ventral edge: smooth continuous line = 0, abruptly tapering or ‘stepped’ margin = 1
9. Mandibular sensory canal: present = 0, absent = 1
10. Mandibular canal exposure: entirely enclosed, opens through lines of pores = 0, mostly enclosed, short sections of open grooves = 1, mostly open grooves, short sections opening through pores = 2, entirely open = 3
11. Mandible: oral sulcus/surangular pit line: present = 0, absent = 1
12. Meckelian bone floors precoronoid fossa: yes = 0, no = 1
13. Meckelian bone ossified in middle part of jaw: yes = 0, little or no ossification = 1
14. Meckelian foramina/ fenestrae, dorsal margins formed by; Meckelian bone = 0, prearticular = 1, infradentary = 2
15. Meckelian foramina/ fenestrae, height: much lower than adjacent prearticular = 0, equal to or greater than depth of adjacent prearticular = 1
16. Adsymphysial lateral foramen present: no = 0, yes = 1 (Note: in this character list we implement a terminology change from "parasymphysial" to "adsymphysial" for the small dermal bone that lies anterior to the coronoid series, and the foramina that are associated with its posterior end. This reflects a conclusion, based on the study of early osteichthyan jaws such as those of porolepiforms and *Psarolepis*, that their tooth-whorl-bearing parasymphysial plates are probably not homologous with the adsymphysial plates of tetrapodomorphs.)
17. Adsymphysial mesial foramen present: no = 0, yes = 1
18. Postsplenial with mesial lamina: no = 0, yes = 1
19. Postsplenial pit line present: yes = 0, no = 1
20. Postsplenial suture with prearticular present: no = 0, yes but interrupted by Meckelian foramina or fenestrae = 1, uninterrupted suture = 2
21. Prearticular sutures with surangular: no = 0, yes = 1
22. Prearticular sutures with mesial lamina of splenial: no, mesial lamina of splenial absent = 0, yes = 1, no, mesial lamina of splenial separated from prearticular by postsplenial = 2
23. Prearticular with longitudinal ridge below coronoids: no = 0, yes = 1
24. Prearticular with mesially projecting flange on dorsal edge along posterior border of adductor fossa: no = 0, yes = 1
25. Prearticular centre of radiation of striations: level with posterior end of posterior coronoid = 0, level with middle of adductor fossa = 1, level with posterior end of adductor fossa = 2
26. Splenial has free ventral flange: yes = 0, no = 1
27. Splenial, rearmost extension of mesial lamina: closer to anterior end of jaw than to adductor fossa = 0, equidistant = 1, closer to anterior margin of adductor fossa than to the anterior end of the jaw = 2

lower jaw dentition

1. Coronoids: at least one has fang pair recognisable because at least twice the height of coronoid teeth: yes = 0, no = 1
2. Coronoids: at least one has fangs recognisable because noticeably mesial to vertical lamina of bone and to all other teeth: yes = 0, no = 1
3. Coronoids: at least one has organised tooth row: yes = 0, no =1
4. Coronoids: at least one carries shagreen: no = 0, yes = 1
5. Coronoids: size of teeth (excluding fangs) on anterior and middle coronoids relative to dentary tooth size: about the same = 0, half height or less = 1
6. Dentary teeth: larger than maxillary teeth = 0, same size as maxillary teeth = 1, smaller than maxillary teeth = 2
7. Dentary with a row of very small teeth or denticles lateral to tooth row: yes = 0, no = 1
8. Adsymphysial tooth plate: present = 0, absent = 1
9. Adsymphysial plate dentition: shagreen or irregular tooth field = 0, organised dentition aligned parallel to jaw margin = 1, no dentition = 2
10. Adsymphsial plate has fang pair: no = 0, yes = 1.
11. Adsymphysial plate has tooth row: no = 0, short tooth row, separated from coronoid tooth row by diastema = 1, long tooth row reaching coronoid = 2
12. Prearticular shagreen field, distribution: gradually decreasing from dorsal to ventral = 0, well defined dorsal longitudinal band = 1, scattered patches or absent = 2

# general skull characters

1. Anterior palatal fenestra: single = 0, double = 1, absent = 2
2. Dorsal fontanelle on snout: absent = 0, present = 1
3. Interpterygoid vacuities: absent = 0, at least 2 x longer than wide = 1, < 2 x longer than wide = 2
4. Intracranial joint: present in dermal skull roof = 0, absent = 1
5. Nature of dermal ornament: tuberculate = 0, fairly regular pit and ridge = 1, irregular = 2, absent or almost absent = 3
6. Nature of ornament: ‘starbursts’ of radiating ornament on at least some bones: no = 0, yes = 1
7. Keyhole-shaped orbits

postcranium

1. Anocleithrum: oblong with distinct anterior overlap area = 0, drop-shaped with no anterior overlap area = 1, absent = 2
2. Cleithrum: ornamented = 0, not ornamented = 1
3. Cleithrum, postbranchial lamina: present = 0, absent = 1
4. Digits: absent = 0, present = 1
5. Humerus: narrow tapering entepicondyle = 0, square or parallelogram-shaped entepicondyle = 1
6. Pectoral process of humerus: absent = 0, present = 1.
7. Proximal limb of oblique ridge of humerus: present, separated from anterior margin of humerus by prepectoral space = 0, absent, replaced by deltopectoral crest = 1.
8. Latissimus dorsi attachment of humerus: diffuse ridged area = 0, distinct process = 1.
9. Foramina piercing oblique ventral ridge of humerus: many = 0, one moderately large foramen in addition to entepicondylar foramen = 1, entepicondylar foramen is the only large opening, other foramina are tiny pinpricks or absent = 2
10. Ilium, iliac canal: absent = 0, present = 1
11. Ilium, posterior process: oriented posterodorsally = 0, oriented approximately horizontally posteriorly = 1
12. Interclavicle: small and concealed or absent = 0, large and exposed = 1
13. Interclavicle shape: ovoid = 0, kite-shaped = 1, with posterior stalk = 2
14. Lepidotrichia in paired appendages: present = 0, absent = 1
15. Posttemporal + supracleithrum: present = 0, absent = 1
16. Radius and ulna: radius much longer than ulna = 0, approximately equal length = 1
17. Ribs, trunk: no longer than diameter of intercentrum = 0, longer = 1
18. Ribs, trunk: all straight = 0, at least some curving ventrally = 1
19. Ribs, trunk: all cylindrical = 0, some or all bear flanges from posterior margin which narrow distally = 1, some or all flare distally = 2
20. Scapular blade: absent = 0, small with narrow top = 1, large with broad top = 2
21. Scapulocoracoid: small and tripodal = 0, large plate pierced by large coracoid foramen = 1, very large plate without large coracoid foramen = 2
22. Subscapular fossa: broad and shallow = 0, deeply impressed posteriorly = 1
23. Squamation: complete body covering of scales, all similar = 0, ventral armour of gastralia = 1
