# Supplementary material for: The smallest known Devonian tetrapod shows unexpectedly derived features
Source: R Soc Open Sci. 2020 Apr 8;7(4):192117. doi: 10.1098/rsos.192117 (PMC7211834; doi:10.1098/rsos.192117)
Supplement: Appendix 2 [file rsos192117supp2.docx]

**APPENDIX 2**

**Data matrix**

1111111111

1111111111222222222233333333334444444444555555555566666666667777777777888888888899999999990000000000

Character: 1234567890123456789012345678901234567890123456789012345678901234567890123456789012345678901234567890123456789

_________________________________________________________________________________________________________________________________

*Acanthostega* 0011111100011110101?0111100010000010000000110?100111110010100110000110011101111110111011011111200111101020201

*Aytonerpeton* 0????0?1000???????????????????121010111????1??0????1110??1?0?1???0?00??1??????0????21??????????00?????100????

*Balanerpeton* 211100011?0210110111011??1010111100001111121??011?11120001120200?101111?011?00220211102111111120011111102?2?1

*Baphetes* 11110??101011111101?01111100011110000111?0????01031???????1??????????????10010?2001111?1111111200??1?1??????1

*Brittagnathus* ????????????????????????????????????????1?0110?10111000??0100110?0100001?101102??????????????????????????????

*Crassigyrinus* 100100?111021011101?0????10?00010000000000200?0103111100001001??1121111?01011011101210?11111111??11111110???1

*Dendrerpeton* 10110001?10210110111111111£10110100011111€2111011?1?111??1111??0?111111?01????22021110211111112001111111022?1

*Densignathus* ????????????????????????????????????????00110?100001000110?0010000000001?101111????31?????1??????????????????

*Diploradus* ?????0???00??01?101??????1?????1????????0001???????11?0??0?001?????1111?110????????2?????????????????????????

*Elginerpeton* ???????????0????????????????????0?0???????1???00000100011000011???110000?001111????11??11??????11????????0?1?

*Elpistostege* 0??11100?001?11?0?????????0???????0???????????0?00????????0??????0??????????????0?1000???????????????????????

*Eoherpeton* 20?10111?01?101111???11?111?0002110?????12?1??011?11110??111?1?12121101101????2?001210???11111200??11111022??

*Eusthenopteron* 0000000000000000000??000000000000000000000100?000000000000000000?0000000100000000000000000000100?0000000000?0

*Greererpeton* 2111£00100021101101001111101000200101111122111010101111000100100?120101101011021111110210111112001111110112?1

*Ichthyostega* 0111111?10001110101?011?1000£0020110010000010?1000010000101001000001100121011120001110?101110011112111111021?

*Megalocephalus* 11111??10?011111101?0111110001111000011110210?010311?2000112110???21110?01011020001111???????????????????????

*Metaxygnathus* ????????????????????????????????????????00100?100101000?10?0010000001001?101111????31????????????????????????

*Panderichthys* 0011000000000000000?0000000000000000000000000?000000000000000000?00000000000000000100000000000???0?0001?101?0

*Parmastega* 0111111100011010000?0?0?1???00010110000000010?1001011?0?100001100000000£21011210001110001????????12??????????

*Pederpes* ?1110001000?10101?1??1??110?001200???????0£??????????????????????????????????????01110?11111112011211110112?1

*Perittodus* ?????????0?????????????????????2?????????0?10?00?????????0????1????01000?1011??????2?????????????????1???????

*Proterogyrinus* 2001000100121011111?11???11?0100000?????1221??011?11111001111?11?12???1?111???2?001210?11111112001?11111022?1

*Sigournea* ?????????????????????????????????????????1211?01031111000010?100?0211001?1?????????11????????????????????????

*Silvanerpeton* 2101000101121011111?11???10???111000011111??0?001?11110??11???00??11111?11????22001210211?11112011211111022?1

*Tantallognathus* ????????????????????????????????????????102?0???0???110??0????101?????????????1????11?????????0??????????????

*Tiktaalik* ?0?111???00?11?0000??00?00?0???0?00?????00000?0000?00?0000??0000???00000?10??00?00100000000000?000?0?010?01?0

*Ventastega* 00?1?11100011110101?001010000?0000100?0000010?1001010001100001100000100111010210101110111??????0011?1????020?

*Whatcheeria* ???10111?0021010101?????11???002??1?????101110000201000000100110102110012101111?001310?10011112011211?111220?

*Ymeria* ??1??????00??????0??0???????00?20?100?0?000?0?10021?000??010??0?00?0100011011110?0?31????????????????????????

£ = 0/1 € = 1/2
